# Platelet sonicates activate hair follicle stem cells and mediate enhanced hair follicle regeneration

**DOI:** 10.1111/jcmm.14873

**Published:** 2019-12-05

**Authors:** Meishu Zhu, Deqiang Kong, Ruiyun Tian, Mengru Pang, Miaohua Mo, Yu Chen, Guang Yang, Hanghang Liu Cheng, Xiaoxuan Lei, Kunwu Fang, Biao Cheng, Yaojiong Wu

**Affiliations:** ^1^ The Graduate School of Southern Medical University Guangzhou China; ^2^ Department of Plastic Surgery General Hospital of Southern Theater Command PLA Guangzhou China; ^3^ Department of Burn & Plastic Surgery the First Affiliated Hospital of Shenzhen University Health Science Center the Second People's Hospital of Shenzhen Shenzhen China; ^4^ The Shenzhen Key Laboratory of Health Sciences and Technology Tsinghua Shenzhen International Graduate School, and Tsinghua‐Berkeley Shenzhen Institute (TBSI) Tsinghua University Shenzhen China; ^5^ Key Laboratory of Tissue Repair and Regeneration of PLA, and Beijing Key Research Laboratory of Skin Injury, Repair and Regeneration The Fourth Medical Center of General Hospital of PLA Beijing China

**Keywords:** androgenic alopecia, hair follicle regeneration, hair follicle stem cells, platelet sonicate, platelet‐rich plasma

## Abstract

An increasing number of studies show that platelet‐rich plasma (PRP) is effective for androgenic alopecia (AGA). However, the underlying cellular and molecular mechanisms along with its effect on hair follicle stem cells are poorly understood. In this study, we designed to induce platelets in PRP to release factors by calcium chloride (PC) or by sonication where platelet lysates (PS) or the supernatants of platelet lysate (PSS) were used to evaluate their effect on the hair follicle activation and regeneration. We found that PSS and PS exhibited a superior effect in activating telogen hair follicles than PC. In addition, PSS injection into the skin activated quiescent hair follicles and induced K15^+^ hair follicle stem cell proliferation in *K14‐H2B‐GFP* mice. Moreover, PSS promoted skin‐derived precursor (SKP) survival in vitro and enhanced hair follicle formation in vivo. In consistence, protein array analysis of different PRP preparations revealed that PSS contained higher levels of 16 growth factors (out of 41 factors analysed) than PC, many of them have been known to promote hair follicle regeneration. Thus, our data indicate that sonicated PRP promotes hair follicle stem cell activation and de novo hair follicle regeneration.

## 1 INTRODUCTION

Androgenic alopecia (AGA), also known as male pattern baldness, is a common disorder that affects men and women. In the past years, preliminary clinical studies have shown that platelet‐rich plasma (PRP) promotes hair regrowth and thus increases hair numbers in AGA patients.^1^, ^2^, ^3^ Recent animal studies suggest that PRP increases the efficiency of hair follicle formation in hair follicle reconstitution experiments and shortens the time of hair formation.^4^, ^5^ Moreover, a recent study suggests that the effective ingredients of PRP can be better released in platelet lysate than conventional calcium‐induced platelet contraction.^6^

Stem cells capable of regenerating the hair follicle reside in the hair follicle bulge, which express specific markers such as K15, CD34 and Lgr5.^7^, ^8^, ^9^ In addition, hair follicle stem cells (HFSCs) contribute to epidermal cells when the skin is wounded.^10^ K14 marks epithelial cells in the basal layer of the epidermis and in the hair follicle, and *K14‐H2B‐GFP* transgenic mice, which express high level of GFP in the nuclei of K14^+^ cells, provide an ideal model to track dynamic cyclic changes of the hair follicle.^11^, ^12^ A variety of growth factors and cytokines have been found to regulate the cyclic activation of HFSCs and their activities in hair follicle regeneration.^8^, ^11^, ^13^

In this study, we examined the effect of PRP preparations processed by supplementation of calcium or by sonication and showed that platelet lysate after sonication exhibited superior effect in activating HFSCs and enhancing hair follicle regeneration than calcium‐induced PRP gel.

## 2 MATERIALS AND METHODS

### 2.1 Preparation of PRP

50 mL of PRP was prepared from 400 mL peripheral blood of a healthy donor according to a method previously described.^14^ PRP was activated either by the addition of 10% calcium chloride (PC) or by sonication. When calcium chloride was added to PRP, the preparation was centrifuged at 2000 *g* for 30 min at 4°C, and the supernatant was collected for subsequent experiments. In the preparation of PRP sonicates (PS), 2 mL of PRP in a centrifuge tube was sonicated for 35 cycles (5 second on and 5 second off for each cycle). In some experiments, the PRP sonicate was further centrifuged at 2000 *g* for 30 min at 4°C to collect the supernatant (PSS).

### 2.2 Mice

C57 male mice (6 weeks old) and BALB/c nu/nu mice (5 weeks old) were purchased from the Laboratory Animal Centre, Guangdong province, China. *K14‐H2B‐GFP* transgenic male mice were purchased from the Jackson Laboratory. The animals were maintained in a temperature controlled environment (20°C ± 1°C) with access to food and water throughout the experiment. All animal procedures were performed with the approval of the Animal Ethics Committee of Tsinghua Shenzhen International Graduate School.

### 2.3 Hair follicle alkaline phosphatase (AP) stain

Full‐thickness dorsal skin tissues of C57 mice were collected and washed with phosphate buffered saline (PBS). Samples were stained with AP Staining Kit (C3206, Beyotime Biotechnology) following the manufacturer's instructions and visualized under microscope (Leica).

### 2.4 Immunofluorescence analysis

The dorsal skin tissues were fixed in 4% paraformaldehyde (PFA) for 12 hours, dehydrated in 30% sucrose for 12 hours and embedded in OCT. Tissue sections (10 μm in thickness) were treated with 0.5% Triton X‐100 (sigma‐Aldrich) for 1 hours, blocked with 3% bovine serum albumin (BSA) and stained with anti‐Ki67 antibody (1:50, Santa Cruz) at 4°C overnight. Samples were then stained with tetraethyl rhodamine isothiocyanate (TRITC)–conjugated secondary antibodies (Jackson ImmunoResearch) and 4′,6‐diamidino‐2‐phenylindole (DAPI), and visualized under confocal laser scanning microscope (FV1000, Olympus).

### 2.5 Culture of SKPs

Skin‐derived precursors (SKPs) were isolated form neonatal mouse skin as described previously.^15^ Briefly, dorsal skin was harvested from neonatal C57BL mice 1–3 days after birth. After treatment with 0.3% Dispase II, the epidermis was manually removed, and the dermis was digested with collagenase I. The dissociated cells were plated in a 10‐cm non‐treated dish using 10 mL Dulbecco's modified Eagle's medium (DMEM)/F12, 3:1 (Gibco) containing B27 (Gibco), 20 ng/mL epidermal growth factor (EGF, PeproTech) and 40 ng/mL basal fibroblast growth factor (bFGF, PeproTech), and incubated in a 37°C, 5% CO_2_ tissue culture incubator.

### 2.6 Cell proliferation assay

Cell proliferation was evaluated using Cell Counting Kit‐8 (CCK‐8).^16^ Cells were starved overnight, then seeded in 96‐well plates (10 000 SKPs per well, or 5000 HaCaT cells per well) and incubated in corresponding culture medium at 37C in 5% CO_2_ for 72 hours, followed by a treatment of 10 μL CCK‐8 for another 3 hours. The culture was then subjected to spectrophotometric analysis with a microplate reader (BioTek) with absorbance at 450 nm.

### 2.7 Hair follicle reconstitution assay

BALB/c nu/nu mice (5 weeks old) were anaesthetized by an intraperitoneal injection of sodium pentobarbital (50 mg/kg). Two symmetrical 5‐mm‐diameter full‐thickness skin wounds were created on the back with a skin biopsy punch as our previously study described.^15^, ^17^, ^18^ We mixed 10^6^ epidermal cells and 2 × 10^6^ dermal cells (per wound) derived from neonatal K14‐H2B‐GFP mice in 20% PSS or equal volume of saline and encapsulated them in 10 μL Matrigel (BD, USA). After incubation for 30 minutes at 37°C, the cells‐Matrigel was implanted into an excisional wound. The wound was then covered with Tegaderm (3M, USA) transparent dressing, which was further covered with self‐adhering elastic bandage. After 3 weeks, the grafts were examined under dissecting microscope (Leica) and two‐photon microscope (Olympus) to logical analysis.

### 2.8 Antibody‐based protein array

PRP samples (PC, PS and PSS) prepared as described above were subjected to antibody‐based protein array analysis using GSM‐CAA‐4000 (RayBiotech) by RayBiotech (Guangzhou), which detect 41 human growth factors as follows: amphiregulin, hepatocyte growth factor (HGF), nerve growth factor (NGF), basic fibroblast growth factor (bFGF), FGF‐4, FGF‐6, FGF‐7/keratinocyte growth factor (KGF), epidermal growth factor receptor (EGFR), EGF, heparin‐binding EGF‐like growth factor (HB‐EGF), insulin‐like growth factor‐binding protein 1 (IGFBP‐1), IGFBP‐2, IGFBP‐3, IGFBP‐4, IGFBP‐6, insulin‐like growth factor 1 (IGF‐1), IGF‐1 receptor (IGF‐1R), IGF‐2, macro phage colony‐stimulating factor (M‐CSF), M‐CSF receptor (M‐CSFR), granulocyte‐macrophage colony‐stimulating factor (GM‐CSF), glial cell line‐derived neurotrophic factor (GDNF), granulocyte colony‐stimulating factor (GCSF), neurotrophin‐3 (NT‐3), NT‐4, placental growth factor (PLGF), platelet derived growth factor (PDGF) subunit AA (PDGF‐AA), PDGF‐AB, PDGF‐BB, alpha‐type PDGF receptor (PDGFRA), PDGFRB, stem cell factor (SCF), SCF receptor (SCFR/CD117/c‐kit), transforming growth factor alpha (TGF‐α), TGF‐β1, TGF‐β2, TGF‐β3, vascular endothelial growth factor A (VEGF‐A), VEGF receptor 2 (VEGFR‐2), VEGFR‐3 and VEGF‐D.

### 2.9 Statistical analysis

All data were expressed as mean ± SD. ANOVA was used for data analysis, and statistical significance was defined as *P* < .05.

## 3 RESULTS

### 3.1 PRP preparations induce hair follicle telogen‐anagen transition

**Figure 1:**
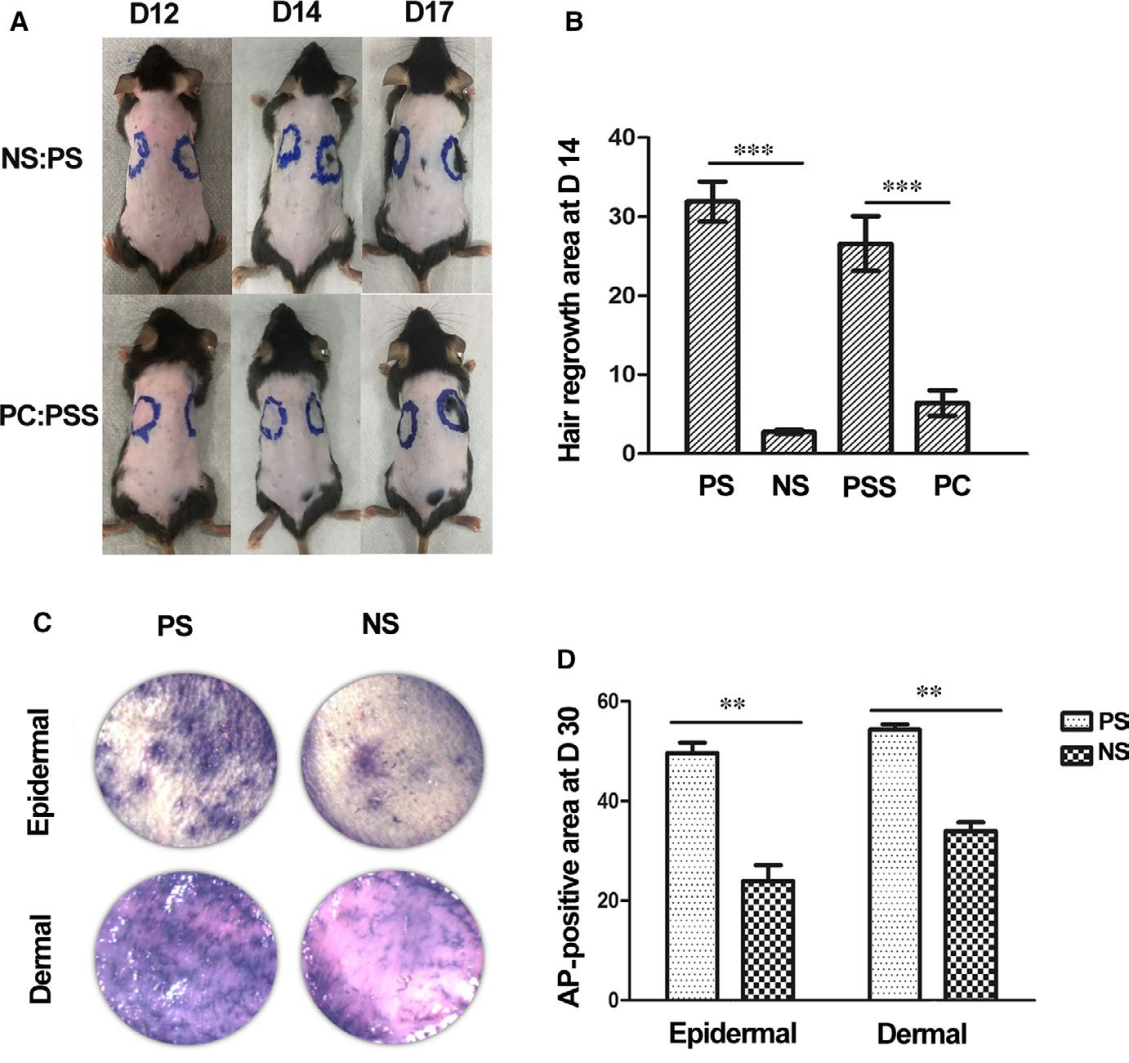
The effect of PRP preparations on hair follicle activation. A, B, After depilation of the dorsal skin of 7‐week‐old C57 mice (hair follicles in telogen phase), equal amounts of saline (NS), calcium‐activated PRP (PC) and PRP sonicate (PS) or the supernatant of PS (PSS) were injected intra‐cutaneously. The injection sites were marked. After 14 days, hair regrowth was observed at the injection sites of PS and PSS, while no evident hair regrowth was found at the injection sites of NS and PC; the areas with hair regrowth were measured (n = 3, **P < .01, ***P < .001). C, D, Thirty days after the injection, the skin tissue of the injection site was excised and subjected to AP stain. Microscopic examination of the epidermal (upper panel) or dermal (lower panel) side detected AP‐positive hair follicles (C) and the areas positive for AP stain were measured (D, n = 3, **P < .01)

To examine the effect of various PRP preparations on hair follicle activation, 7‐week‐old C57 mice (hair follicles in telogen phase) received intra‐cutaneous injection of saline (NS), calcium‐activated PRP (PC), PRP sonicate (PS) or the supernatant of PS (PSS). After 14 days, hair regrowth was observed at the injection sites of PS and PSS, while sparse hair regrowth was found at the injection sites of NS and PC. Quantitation of the hair regrowth areas showed that PSS induced enhanced hair regrowth compared to NS and PC (Figure 1A,B, *P* < .01). Thirty days after the injection, the skin tissue of the injection site was excised and subjected to AP stain; more AP‐positive hair follicles in anagen phase were detected in the skin receiving PS injection (Figure 1C,D). These results indicate that PRP sonicates have enhanced effect in inducing hair follicle telogen‐anagen transition (TAT).

### 3.2 Sonicated PRP activates hair follicle stem cells

**Figure 2:**
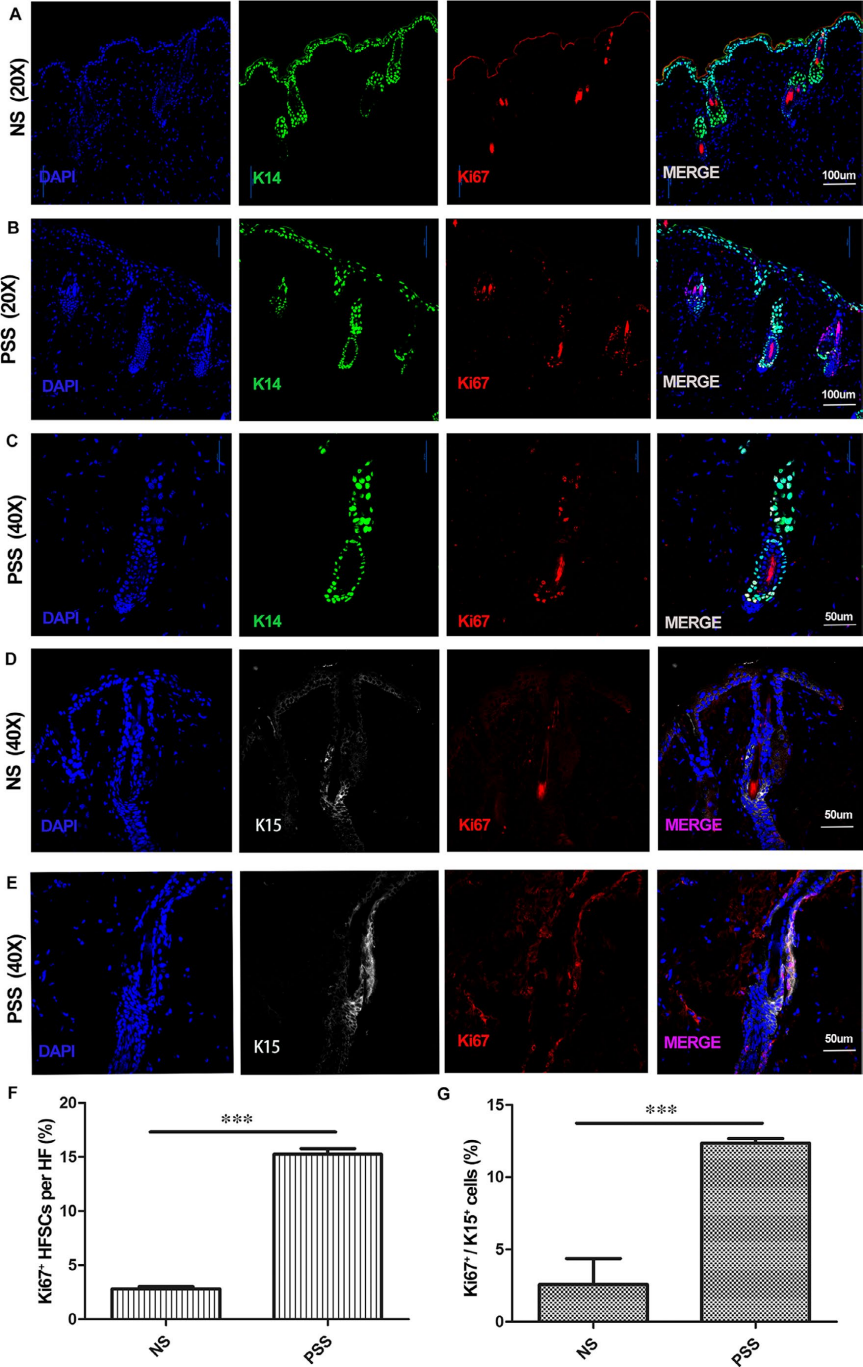
The effect of PRP sonicate on hair follicle stem cell activation. *K14‐H2B‐GFP* mice (7 weeks old) received skin injection of the supernatant of PRP sonicate (PSS) or saline (NS). Five days after the injection, fluorescence microscopy showed an increase in the number of GFP‐positive K14 cells in the hair follicle at the injection site of PSS, compared to the hair follicle at the saline injection site. Immunofluorescence (IF) stain for Ki67 showed an increased number of proliferating K14‐GPF cells in the hair follicle after PSS injection (A‐C). IF stain for K15 and Ki67 showed increases in the number of K15^+^ hair follicle stem cells (HFSCs) and in the percentage of Ki67^+^ K15^+^ HFSCs after PSS treatment (D‐G, n = 3, 10 hair follicles in full size per tissue sample were measured, ***P < .001)

To investigate the mechanisms underlying PRP‐induced hair follicle TAT, *K14‐H2B‐GFP* mice (7 weeks old) whose K14^+^ cells expressed high level GFP in the nuclei received skin injection of PSS or saline. Five days after injection, confocal microscopy showed a dramatic increase in the number of K14‐GFP‐positive cells in the hair follicle at the injection site of PSS, compared to the saline injection site. In consistence, Ki67 stain showed increased numbers of proliferating K14‐GFP cells in the hair follicle after PSS injection (Figure 2A‐C). To examine alterations of HFSCs after the treatments, we performed dual immunofluorescence stain for K15^+^ cells and Ki67, and observed a significant increase in the number of K15^+^ cells in the hair follicle after PSS treatment compared to saline treatment, which was associated with an increase in the number of dividing K15^+^ cells which expressed Ki67 (Figure 2D‐G). These results indicate that factors released from platelets activate quiescent HFSCs, and promotes their proliferation, resulting in hair follicle TAT.

### 3.3 PRP promotes cell survival and proliferation

**Figure 3:**
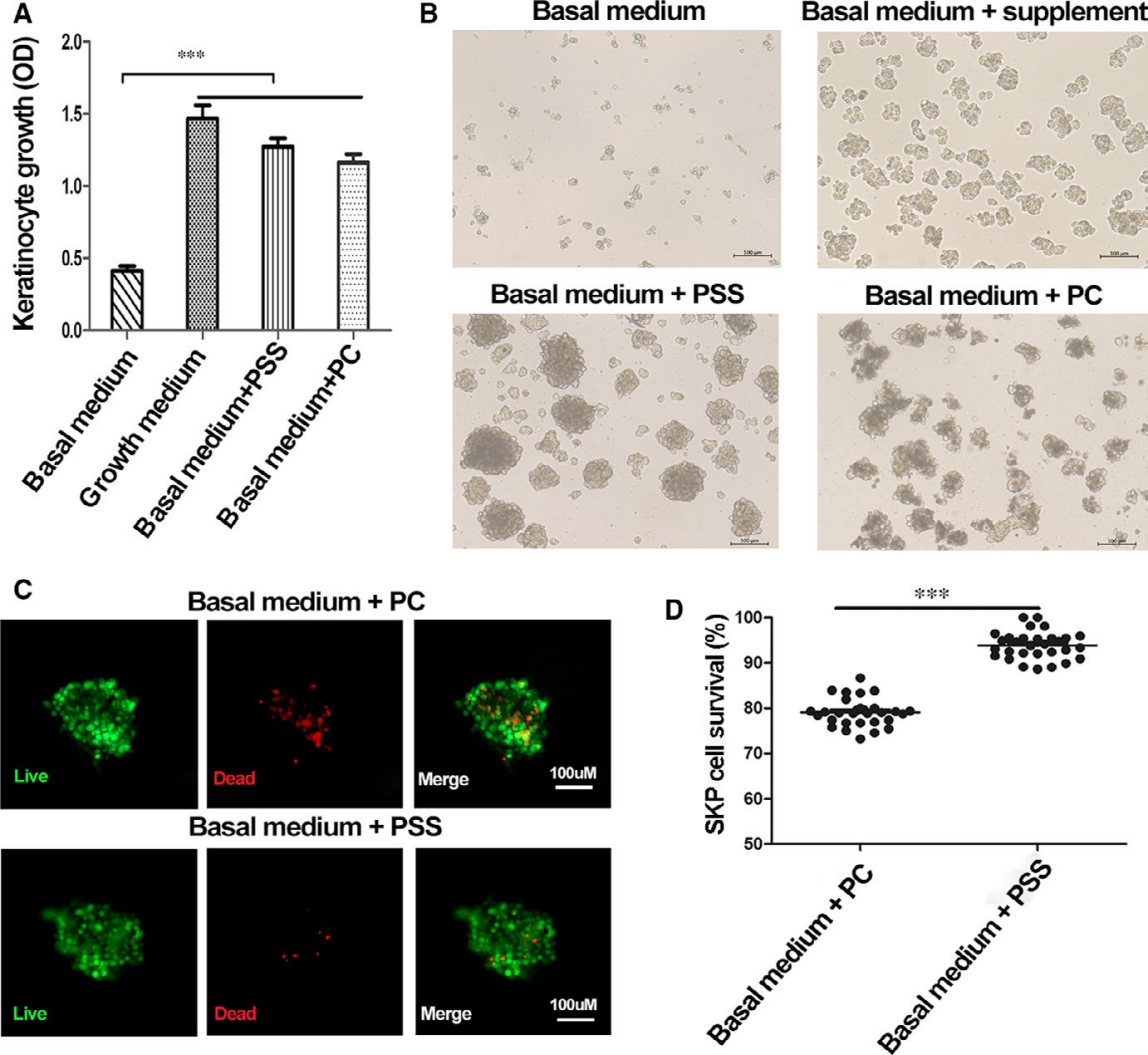
The effect of PRP preparations on skin cell proliferation and survival. Human keratinocytes HaCaT and mouse SKPs were cultured in basal medium with supplementation of PRP activated by calcium (PC), the supernatant of PRP sonicates (PSS) or regular growth supplements (growth medium for positive control), respectively. A, HaCaT cells were incubated for 3 days and subjected to CCK‐8 analysis. ***P < .001. B‐D, after incubation for 2 days, SKPs in basal medium formed small aggregates; in regular growth medium (basal + supplement) cells formed large aggregates (B); in culture supplemented with PSS cell formed large aggregates with healthy appearance, while in culture supplemented with PC many cells in the aggregates appeared dead (B). Cell aggregates from PSS and PC cultures were stained using a Live/Dead cell viability kit where viable cells were stained green and dead cells were shown in red (C), and the number of viable and dead cells was counted resulting in cell viability (D, ***P < .001). Triple wells were used for cell culture experiments. Experiments were repeated three times, and representative results were shown

To examine the effect of PRP on skin cells, we supplemented PRP preparations to human keratinocyte (HaCaT) and mouse dermal stem cell (SKP) cultures. Both PC and PSS significantly promoted the proliferation of keratinocytes (Figure 3A). In the absence of regular supplement, SKPs formed small cell aggregates in basal medium compared to SKPs in regular growth medium (basal medium plus regular supplement) (Figure 3B). Notably, supplementation of PC to basal medium resulted in the formation of large cell aggregates, but a considerable fraction of cells died after 2 days (79 ± 3.0% cell viability as determined by Live/Dead cell viability assay); when PSS was added to the culture, however, SKPs formed large aggregates with healthy morphology (93 ± 2.9% cell viability, *P* < .001 compared to PC) (Figure 3B‐D). The results indicated that PSS exhibited enhanced effect in promoting SKP survival.

### 3.4 PRP sonicates promote hair follicle formation

**Figure 4:**
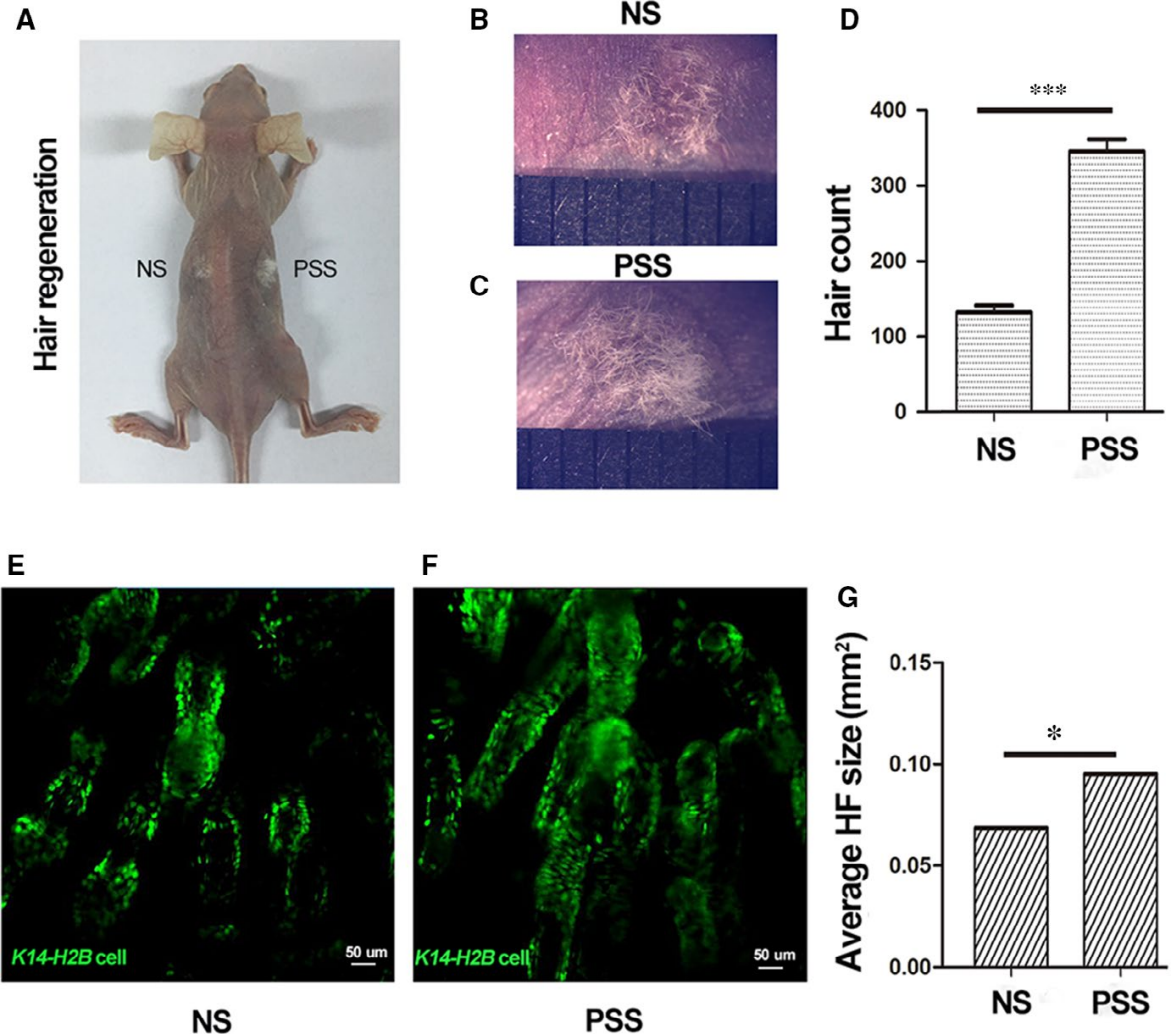
The effect of PRP sonicate on hair follicle reconstitution. Epidermal and dermal cells derived from neonatal *K14‐H2B* mice were seeded into dorsal excisional wounds of nude mice in combination with saline (NS) or the supernatant of PRP sonicates (PSS) (n = 3). Three weeks after transplantation, macroscopic appearance of reconstituted skin showed more hairs at the graft sites with PSS than with NS (A‐C), and the number of the hairs was counted (D, ***P < .001). Two‐photon microscopic analysis of the skin showed larger GFP‐positive hair follicles in PSS‐treated grafts than NS‐treated grafts (E‐G, n = 3, 20 hair follicles per graft were measured, *P < .05). Representative images were shown

To examine the effect of PRP in hair follicle formation, we performed hair follicle reconstitution assay. 10^6^ epidermal cells and 2 × 10^6^ dermal cells (per wound) derived from neonatal *K14‐H2B‐GFP* mice in 20% PSS or equal volume of saline were implanted into 2.5 mm excisional wounds. Three weeks after transplantation, sites of grafts containing PSS generated more hairs than sites of grafts containing control saline (Figure 4A‐D). In addition, two‐photon microscopy showed the PSS‐supplemented grafts generated larger hair follicles than saline‐supplemented grafts (Figure 4D‐E). These results indicate that PRP sonicates enhance de novo hair regeneration and hair growth.

### 3.5 PRP sonicates contain higher levels of growth factors

**Figure 5:**
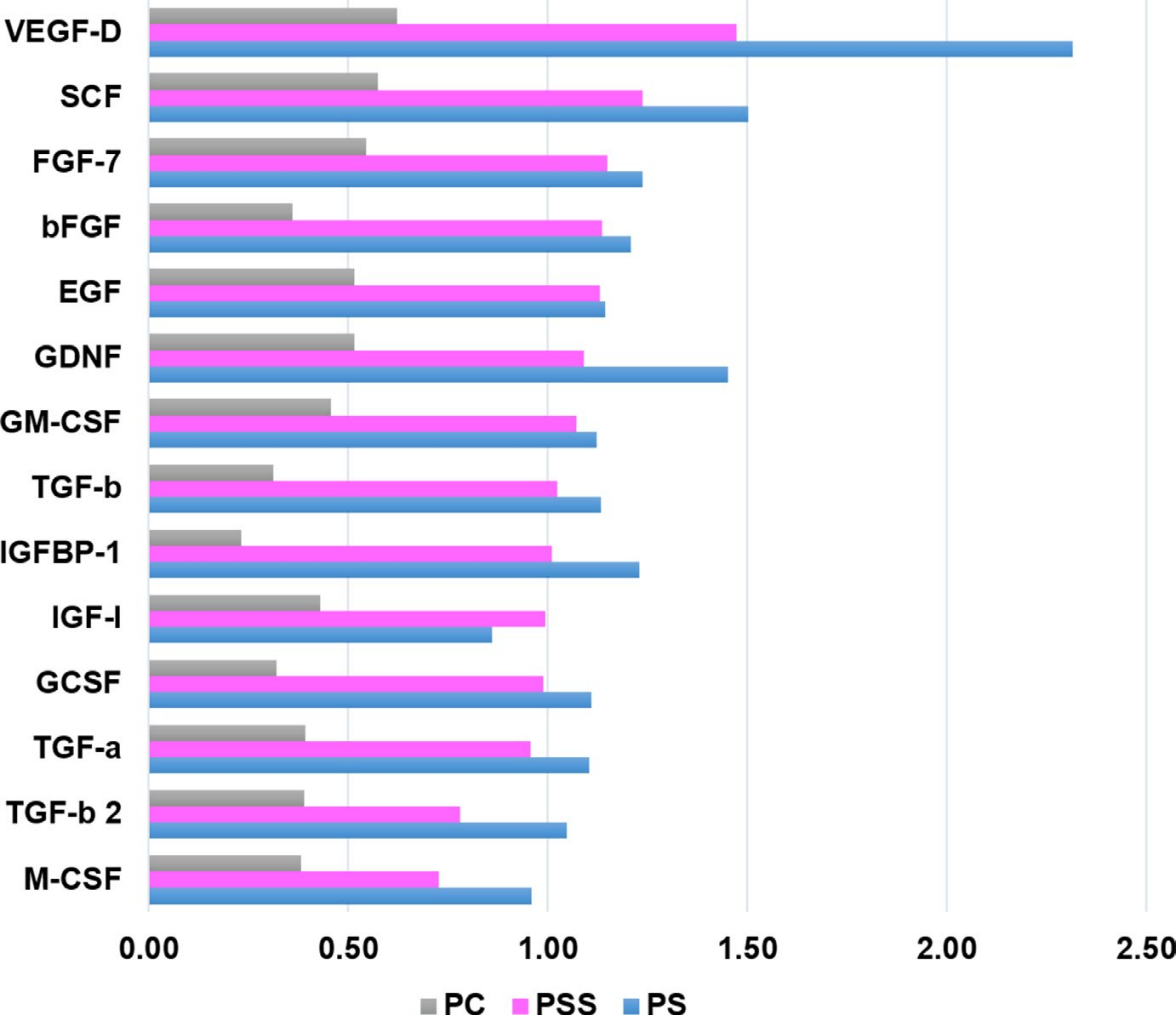
Protein array analysis of growth factors in PRP preparations. Antibody‐based protein array analysis of 41 growth factors revealed 16 growth factors with higher levels (≥2‐fold) in the supernatant of PRP sonicate (PSS) than in calcium‐activated PRP (PC). PS, PRP sonicates (without centrifugation)

To understand why PRP sonicates mediated better effect in inducing hair follicle activation and SKP survival, we analysed the level of growth factors in different PRP preparations (PC, PS and PSS) via antibody‐based protein array. The results indicated that out of 41 growth factors analysed, 16 factors showed higher levels (≥2 folds) in PSS than in PC, which included VEGF‐D, PDGF‐AA, PDGF‐BB, PDGF‐AB, HGF, SCF, GDNF, FGF‐4, FGF‐7, bFGF and IGF‐1 (Figure 5). The results suggest that sonication better releases growth factors from platelets in PRP compared to conventional calcium‐induced platelet contraction.

## 4 DISCUSSION

Platelets are the first cell type to arrive at the site of tissue injury and are actively involved in the healing process. They play roles in homeostasis through diverse mechanisms involving cell membrane adherence, aggregation, clot formation and release of substances that promote tissue repair and regeneration.^19^ PRP has been shown efficacy in enhancing the repair of various tissue injuries such as bone, tendon, ligament and muscle in preclinical studies and shown promising therapeutic results in several preliminary clinical observations.^19^, ^20^, ^21^ Recently, PRP was found to enhance the healing of complex wounds in elderly patients in a preliminary study.^21^ In addition, PRP has been used in several recent observations in combination with materials known to enhance tissue repair. For example, a combination of PRP and collagen matrix was found to enhance chondrogenic and osteogenic differentiation of adipose‐derived stem cells in vitro.^22^ Notably, PRP has been used together with hyaluronic acid to treat 15 patients with lower‐extremity wound with post‐traumatic bone exposure 23 and a patient with severe hidradenitis suppurativa,^24^ respectively, and showed beneficial outcome. In another study of 10 patients, autologous fat was used in combination with PRP in breast reconstruction and showed improvement in the maintenance of fat volume after surgery.^25^ These results may suggest a synergistic effect of PRP in tissue repair when used in combination with supporting materials.

Previous studies have found that PRP increases hair regrowth in mice and AGA patients.^1^, ^2^, ^5^, ^6^, ^26^, ^27^ In a randomized, evaluator‐blinded, placebo‐controlled trial of 23 male patients with male pattern hair loss, local injection of PRP showed enhancement in hair regrowth.^2^ These studies suggest an effect of PRP on HFSCs. In this study, we provided evidence to show that the supernatant of PRP sonicate (PSS) induced HFSC activation and proliferation, resulting in hair follicle regrowth.

Recent studies showed that transplantation of autologous cells prepared from the hair follicle could form de novo hair follicles in AGA patients.^28^, ^29^ Based on our findings and previous studies, PRP may be contained in grafts for hair genesis to enhance graft survival and improve hair regeneration.^30^

We found that sonicated PRP exhibited an enhanced effect than calcium‐activated PRP in inducing hair follicle regrowth and in promoting SKP survival. Currently, the growth promoting effect of PRP on the hair follicle has been attributed to its growth factors which are stored in the platelets. The conventional method to release growth factors from the platelets is to induce platelet contraction by calcium which activate thrombin.^4^, ^31^ In this study, we found that sonication of PRP which broke platelets into tiny pieces better released growth factors, as evidenced by growth factor array analysis. Sixteen growth factors of 41 growth factors analysed showed higher levels in sonicated PRP than in calcium‐treated PRP, and several of them have been known to regulate hair follicle cell activities such as FGF7, VEGF, PDGF‐BB, HB‐EGF and TGF‐β.^26^, ^32^, ^33^, ^34^, ^35^, ^36^ Our results are in agreement with a previous study, where sonicated PRP was found to contain higher levels of TGF‐β1, VEGF and PDGF‐BB (of four growth factors analysed by ELISA).^6^ It is likely that platelet contraction allows a release of a fraction of growth factors, and some growth factors such as HB‐EGF which are in association with cell membrane 33, 37 are better released by sonication.

## 5 CONCLUSION

This study has provided convincing data to show that PRP preparations activate HFSCs and promote hair follicle regeneration, and platelet sonicates contain high levels of numerous growth factors and exhibit superior effects in promoting SKP survival, suggesting a better preparation for PRP therapy.

## CONFLICT OF INTEREST

All authors declare that they have no competing interests.

## AUTHOR CONTRIBUTIONS

Meishu Zhu and Deqiang Kong designed and performed experiments, and analysed data. Ruiyun Tian prepared *K14‐H2B‐GFP* mice. Guang Yang, Miaohua Mo, Yu Chen, Mengru Pang, Hanghang Liu Cheng, Xiaoxuan Lei and Kunwu Fang performed experiments and analysed data. Yaojiong Wu and Biao Cheng designed experiments, provided materials and wrote the manuscript.

## Data Availability

The data are available from the corresponding author upon reasonable request.
