# Corrigendum

**DOI:** 10.1111/jcmm.15442

**Published:** 2020-07-09

In the article,^1^ the authors have reported a correction on the first affiliation.

The affiliation reads:

^1^The Graduate School of Southern Medical University, Guangzhou, China

The affiliation should have read:

^1^The First School of Clinical Medicine, Southern Medical University, Guangzhou, China
